# COVID-19 and tuberculosis coinfection: An overview of case reports/case series and meta-analysis of prevalence studies

**DOI:** 10.1016/j.heliyon.2023.e13637

**Published:** 2023-02-10

**Authors:** Parham Daneshvar, Bahareh Hajikhani, Fatemeh Sameni, Negin Noorisepehr, Fereshteh Zare, Nazila Bostanshirin, Shahrooz Yazdani, Mehdi Goudarzi, Saba Sayyari, Masoud Dadashi

**Affiliations:** a School of Medicine, Alborz University of Medical Sciences, Karaj, Iran; b Department of Microbiology, School of Medicine, Shahid Beheshti University of Medical Sciences, Tehran, Iran; c Department of Microbiology, Faculty of Medicine, Shahed University, Tehran, Iran; d Department of Biotechnology, School of Medicine, Alborz University of Medical Sciences, Karaj, Iran; e Department of Microbiology, School of Medicine, Alborz University of Medical Sciences, Karaj, Iran; f Department of Cardiology, School of Medicine, Alborz University of Medical Sciences, Karaj, Iran; g Cardiovascular Research Center, Shahid Rajaei Educational and Medical Center, Alborz University of Medical Sciences, Karaj, Iran; h Shahid Beheshti University of Medical Sciences, Imam Hussein Hospital, Tehran, Iran; i Neonatal Health Research Center, Research Institute for Children's Health, Shahid Beheshti University of Medical Sciences, Tehran, Iran; j Non-Communicable Diseases Research Center, Alborz University of Medical Sciences, Karaj, Iran

**Keywords:** COVID-19, *Mycobacterium tuberculosis*, Coinfection, Systematic review, Meta-analysis

## Abstract

**Background and aim:**

Coronavirus disease 2019 (COVID-19) coinfection with other respiratory pathogens poses a serious concern that can complicate diagnosis, treatment, and prognosis. Since COVID-19 and tuberculosis are both severe respiratory infections, their symptoms may overlap and even increase mortality in case of coinfection. The current study aimed to investigate the coinfection of tuberculosis and COVID-19 worldwide through a systematic review and meta-analysis.

**Methods:**

A systematic literature search based on the Systematic Reviews and Meta-Analyses” (PRISMA) was performed on September 28, 2021, for original research articles published in PubMed, Web of Science, and Embase databases from December 2019 to September 2021 using relevant keywords. Data analysis was performed using Stata 14 software.

**Results:**

The final evaluation included 18 prevalence studies with 5843 patients with COVID-19 and 101 patients with COVID-19 and *Mycobacterium tuberculosis* (*M. tuberculosis*). The prevalence of tuberculosis infection was 1.1% in patients with confirmed COVID-19. This coinfection among patients with COVID-19 was 3.6% in Africa, 1.5% in Asia, and 1.1% in America. Eighteen case reports and 57 case series were also selected. Eighty-nine adults (67 men and 22 women) with a mean age of 45.14 years had concurrent infections with tuberculosis. The most common clinical manifestations were fever, cough, and weight loss. A total of 20.83% of evaluated patients died, whereas 65.62% recovered. Lopinavir/ritonavir was the most widely used antiviral drug for 10.41% of patients.

**Conclusion:**

COVID-19 has a low prevalence of tuberculosis coinfection, but it remains a critical issue, especially for high-risk individuals. The exact rate of simultaneous tuberculosis in COVID-19 patients could not be reported since we didn't have access to all data worldwide. Therefore, further studies in this field are strongly recommended.

## 1 Introduction

The emergence of severe acute respiratory syndrome coronavirus 2 (SARS-CoV-2) causing the coronavirus disease 2019 (COVID-19) in China, which was first identified at the end of 2019, sparked a global outbreak of the disease and is a major public health concern [1]. By January 2022, approximately 5.5 million people had lost their lives due to COVID-19 [2]. Droplets and contact with an infected person easily spread the virus. Symptoms of the disease are usually fever and cough, but it can also cause symptoms of muscle pain (myalgia), diarrhea, vomiting, and shortness of breath (dyspnea) in patients [3]. It puts the elderly and people with underlying diseases at greater risk for severe forms of the disease and mortality [4]. Accurate identification of COVID-19 is crucial because it leads to the control and prevention of infection in the community and appropriate and timely treatment measures in patients [5]. Coinfection in COVID-19 is an essential issue because it may create problems for the medical staff in diagnosing, treating, and prognosis of COVID-19. Therefore, concomitant infections must be carefully identified [6,7]. One of the pathogens that can cause coinfection in COVID-19 is *Mycobacterium tuberculosis* (*M. tuberculosis*), which is the cause of tuberculosis [8]. Tuberculosis is an infectious disease that, like COVID-19, is transmitted through the respiratory tract and affects the lungs [9]. Concomitant COVID-19 and tuberculosis infection make the diagnosis and treatment of COVID-19 more challenging, increasing mortality and non-recovery risks [10,11]. Based on WHO reports, due to the COVID-19 pandemic, years of progress in providing essential TB services to patients and reducing the burden of TB disease have been reversed [12]. Despite successes in some countries and regions, global TB targets are largely off track. Globally, there has been a large drop in the number of newly diagnosed and reported cases of TB. In 2020, TB cases fell from 7.1 million in 2019 to 5.8 million [12]. 93% of this reduction was accounted for by 16 countries, with India, Indonesia, and the Philippines suffering the most [13]. The reduction in the number of people treated for drug-resistant tuberculosis (−15%) and TB preventive treatment (−21%) is another impact, and a decline in global spending on TB diagnostic, treatment, and prevention services (from US$ 5.8 billion to US$ 5.3 billion) [13]. In such a situation, the co-occurrence of COVID-19 in TB patients can pose more risks. Numerous studies have been performed on coinfection with COVID-19 and tuberculosis since the onset of the new coronavirus pandemic worldwide [[14], [15], [16]]. However, a systematic review study has yet to be conducted to summarize the complete information on these patients, including symptoms, medications, laboratory findings, and chest CT scans. Given this issue's importance, this study's purpose was to systematically review articles related to coinfection with COVID-19 and *M. tuberculosis* (Active or Latent) and then to analyze the data.

## 2 Methods

We conducted the current review and meta-analysis under the “Preferred Reporting Items for Systematic Reviews and Meta-Analyses” (PRISMA) [17].

### 2.1 Search strategy and study selection

PubMed (MEDLINE), Web of Science, and Embase, the most important electronic databases, were searched on September 28, 2019, to identify relevant studies published in English between December 2019 and September 2021. The following keywords were used: (“COVID-19” [Title/Abstract] OR “novel coronavirus 2019” [Title/Abstract] OR “2019 ncov” [Title/Abstract] OR “nCoV” [Title/Abstract] OR “severe acute respiratory syndrome coronavirus 2” [Title/Abstract] OR “SARS-CoV-2” [Title/Abstract]) AND (“Tuberculosis” [Title/Abstract] OR “*Mycobacterium tuberculosis*” [MeSH Terms] OR “TB” [Title/Abstract] OR “*M. tuberculosis*” [Title/Abstract]). A review of references within covered studies was also done to ensure that relevant publications were noticed. Two investigators independently checked this process. The PICO algorithm was adopted to define inclusion and exclusion criteria for study selection. Accordingly, we evaluated the data on P (Patient, Population, or Problem) = patients with COVID-19, I (Intervention or exposure) = *M. tuberculosis* infection, C (Comparison) = not applicable, and O (Outcome) = coinfection outcome of COVID-19 and tuberculosis. All clinical studies investigating the presence of *M. tuberculosis* infection in patients with COVID-19 were selected, articles that reported only the prevalence of COVID-19 or tuberculosis alone, review articles, abstracts presented in conferences, and duplicate studies were excluded. Relevant prevalence studies, case reports, and case series were further evaluated. Afterward, two investigators screened the titles and abstracts of all selected papers. Next, all selected articles were reviewed in their entirety. Review authors discussed and resolved discrepancies in the article selection or technical uncertainties.

### 2.2 Data extraction

A data extraction form was developed by extracting the first author's name, the year of publication, the type of study, the country where the study took place, the age and gender of patients, the number of confirmed COVID-19 patients, and the number of tuberculosis coinfected patients. Two authors independently recorded the data to avoid any bias.

### 2.3 Statistical analysis

Stata software was used for the statistical analysis (version 14, IC; STATA Corporation, College Station, TX, USA). The pooled proportion of coinfected patients was estimated. The pooled frequency with 95% confidence intervals (CI) was assessed. Statistical heterogeneity was assessed using the I2 method. Cochran's Q and the I2 statistic were used to determine between-study heterogeneity.

## 3 Results

**Fig. 1:**
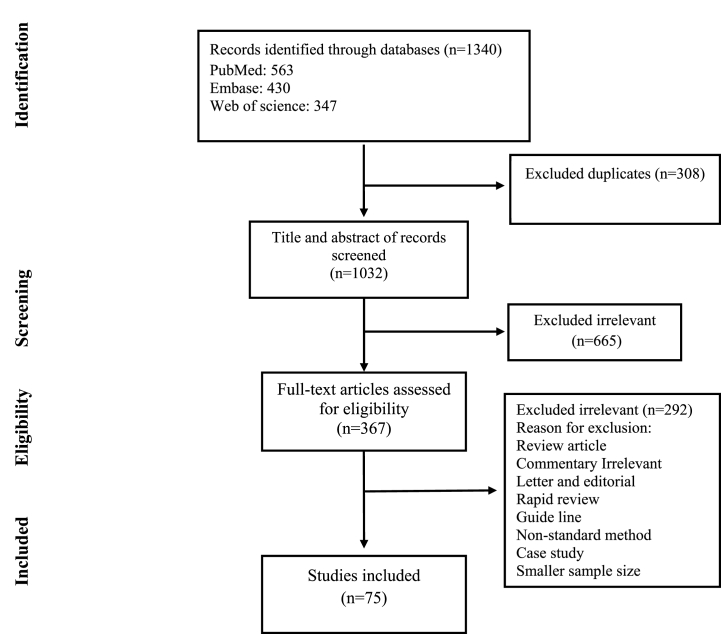
Flow chart of study selection for inclusion in the systematic review and meta-analysis.

Initial searching yielded 1340 articles; duplicates were removed to leave 1032 for the secondary screening. A total of 665 articles were excluded after the title and abstract screening. Review articles, duplicate papers, systematic reviews, unrelated articles, and articles published in languages other than English were excluded from the review process. After reviewing the full text of the studies, eventually, 75 studies met the inclusion criteria and were included in the final analysis (Fig. 1). Eighteen prevalence studies and 75 case reports/series were included in the final selection. The characteristics of the articles are summarized in Table 1, Table 2.

**Table 1:** Characteristics of included prevalence studies.

First author	Published time	Country	Patients with COVID-19	Patients with COVID-19-TB	Mean age	Male/Female
Li [1]	June 2020	China	18	1	nr	nr
Xu [2]	Apr 2020	China	23	1	nr	nr
Himwaze [3]	May 2021	Africa	29	3	44	2 M/1 F
Goel [4]	March 2021	India	35	1	nr	nr
Medina [5]	July 2021	Guatemala	44	1	nr	nr
Niang [6]	Oct 2020	Senegal	47	3	nr	nr
Guan [7]	March 2020	China	53	2	nr	nr
Sun [8]	Aug 2020	USA	63	1	nr	nr
Lai [9]	May 2020	China	110	1	nr	nr
Li [10]	Feb 2021	China	125	3	nr	nr
Van der Zalm [11]	Jun 2021	South Africa	159	7	nr	nr
Sahu [12]	Jan 2021	India	218	1	nr	nr
Rosenberg [13]	March 2020	USA	229	1	84	1 M
Lu [14]	May 2020	China	270	16	nr	nr
Mithal [15]	Feb 2021	India	401	2	nr	nr
Mafort [16]	Apr 2021	Brazil	447	4	nr	nr
Gupta [17]	Oct 2020	India	1073	22	36	nr
Lagrutta [18]	Jan 2021	Argentina	2499	31	nr	nr
**Total**			**5843**	**101**

**Table 2:** Characteristics of included case report/case series.

First author	Published time	Country	patients with COVID-19	patients with COVID-19 -TB	Mean age	Gender
Widiasari [19]	Nov 2020	Indonesia	2	1	43	1 F
Patil [20]	Jun 2021	India	1	1	75	1 M
Agada [21]	Feb 2021	Nigeria	1	1	nr	1 M
Adekanmi [22]	Nov 2020	Nigeria	3	2	65	2 M
Aissaou [23]	Aug 2021	French	1	1	30	1 M
Singh [24]	March 2021	India	9	3	nr	nr
Sasson [25]	Dec 2020	USA	1	1	44	1 M
Bouaré [26]	July 2020	Morocco	1	1	32	1 F
Castillo [27]	Aug 2020	Germany	1	1	64	nr
Dyachenko [28]	May 2021	Ukraine	1	1	44	1 F
Fahad Faqihi [29]	July 2020	Saudi Arabia	1	1	60	1 M
Essajee [30]	Aug 2020	South Africa	1	1	2 years and 7 months	1 F
Ata [31]	Aug 2020	India	1	1	28	1 M
Gbenga [32]	Oct 2020	Nigeria	2	2	31	2 M
Gennaro [33]	March 2021	Italy	4	4	nr	nr
Ghodrati Fard [34]	Feb 2021	Iran	3	3	35.6	3 M
He [35]	Sep 2020	China	3	3	56.3	3 M
Ridgway [36]	Jul 2020	USA	5	1	51	1 F
Orozco [37]	Nov 2020	Mexico	1	1	41	1 M
Farias [38]	Oct 2020	Brazil	2	2	41	2 M
Lopinto [39]	Sep 2020	France	1	1	58	1 M
Musso [40]	Jan 2021	Moldova	1	1	45	1 M
Luciani [41]	Oct 2021	Italy	1	1	32	1 F
Alkhateeb [42]	Nov 2020	Qatar	1	1	28	1 M
Khayat [43]	Jan 2021	Saudi Arabia	1	1	40	1 F
Elziny [44]	May 2021	Nepal	1	1	29	1 M
Baskara [45]	Feb 2021	Indonesia	1	1	42	1 M
Mulale [46]	March 2021	South Africa	1	1	3 months	1 M
Rivas [47]	Oct 2020	Panama	2	2	41	2 M
Ntshalintshali [48]	Mar 2021	South Africa	1	1	65	1 M
Çınar [49]	Oct 2020	Turkey	1	1	55	1 M
Goussard [50]	Aug 2020	South Africa	1	1	3 years	1 F
Goussard [51]	Jul 2020	South Africa	1	1	2 year and 5 months	1 M
Pinheiro [52]	Oct 2020	Brazil	1	1	68	1 M
Saraceni [53]	Jun 2020	Italy	1	1	59	1 M
Sarınoğlu [54]	July 2020	Turkey	2	2	58	2 F
Segrelles-Calvo [55]	Apr 2021	Spain	1	1	58	1 M
Shabrawishi [56]	Apr 2021	Saudi Arabia	7	7	35	4 M/3 F
Gerstein [57]	Feb 2021	El Salvador	1	1	49	1 M
Dakhlia [58]	Sep 2021	Qatar	1	1	34	1 M
Singh [59]	Aug 2020	India	1	1	76	1 F
Pillay [60]	Jan 2021	Durban	1	1	44	1 F
Srivastava [61]	May 2021	India	7	7	60	4 M/3 F
Vilbrun [62]	Nov 2020	USA	1	1	26	1 M
Stjepanović [63]	Jan 2021	Serbia	1	1	27	1 M
Subramanian [64]	March 2021	India	1	1	30	1 F
Tham [65]	Nov 2021	Bangladesh	1	1	40	1 M
Tham [65]	Nov 2020	India	3	3	29	3 M
Cutler [66]	Jul 2020	USA	1	1	61	1 M
Sarma [67]	Nov 2020	India	1	1	53	1 F
Verma [68]	Nov 2020	India	4	4	35.5	3 M/1 F
Wong [69]	Nov 2020	Singapore	1	1	47	1 M
Ortiz-Martinez [70]	Jun 2021	Colombia	1	1	34	1 F
Yadav [71]	Aug 2020	India	1	1	43	1 M
Yao [72]	Nov 2021	China	3	3	50	3 M
Zhang [73]	Sep 2020	China	7	1	75	1 M
Yousaf [74]	Sep 2020	Nepal	2	2	33	2 M
Yousaf [74]	Sep 2020	India	2	2	38.5	2 M
Yousaf [74]	Sep 2020	Bangladesh	2	2	35	2 M
ВОРОБЬЕВА [75]	May 2021	Russia	1	1	55	1 M
**Total**			**114**	**96**		

### 3.1 Prevalence studies

**Fig. 2:**
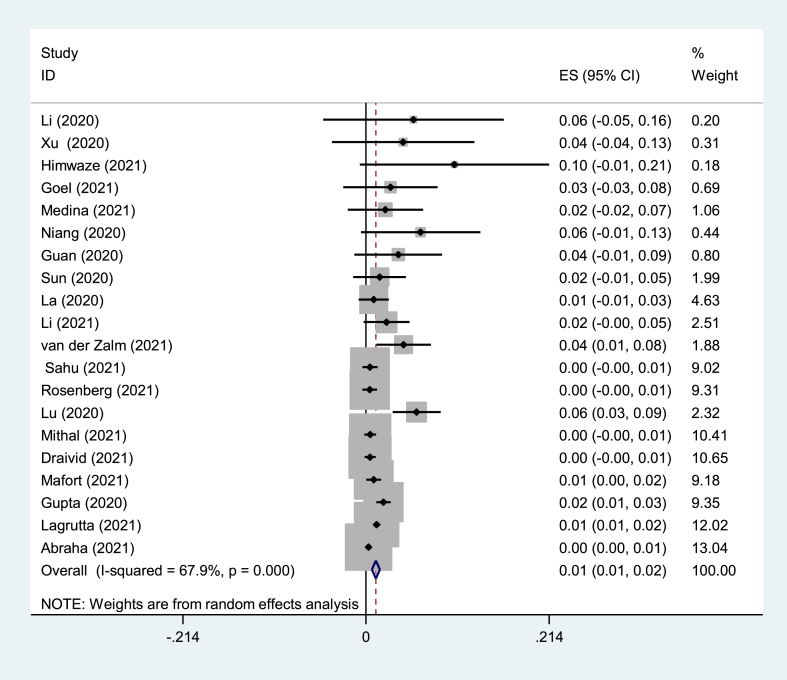
Forest plot of the meta-analysis on the prevalence of TB among patients with COVID-19.

**Fig. 3:**
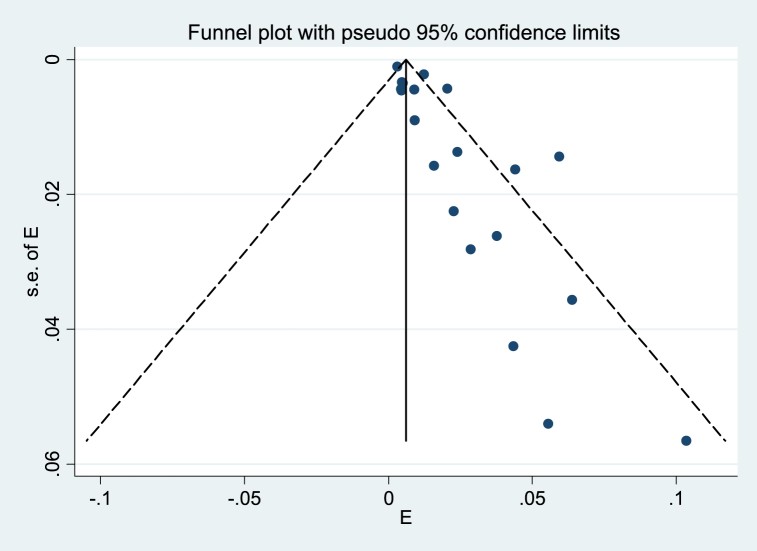
Funnel plot of the meta-analysis on the prevalence of TB among patients with COVID-19. Solid circles represent each study in the meta-analysis.

**Fig. 4:**
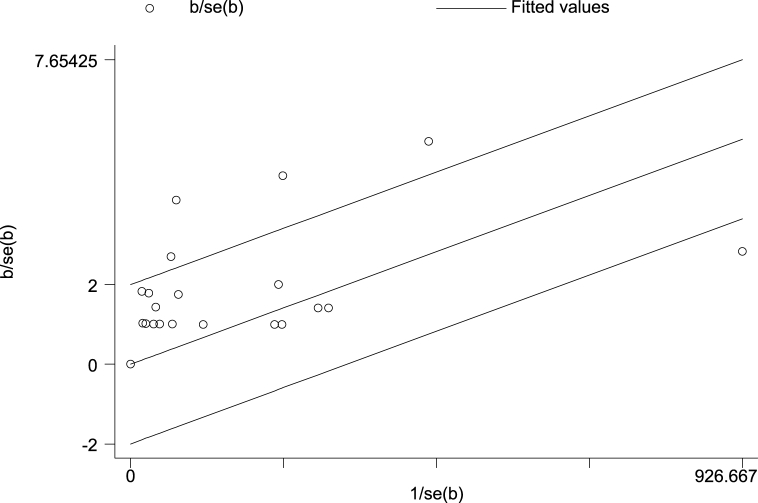
Galbraith of the meta-analysis on the prevalence of TB among patients with COVID-19.

**Fig. 5:**
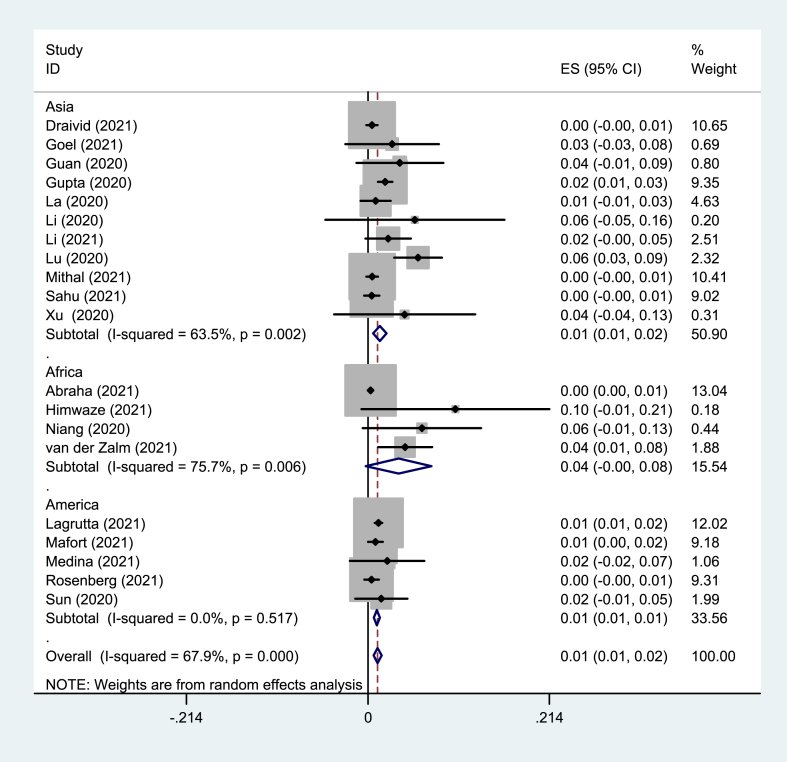
Forest plot of the meta-analysis on the prevalence of TB among patients with COVID-19 based on continents.

**Fig. 6:**
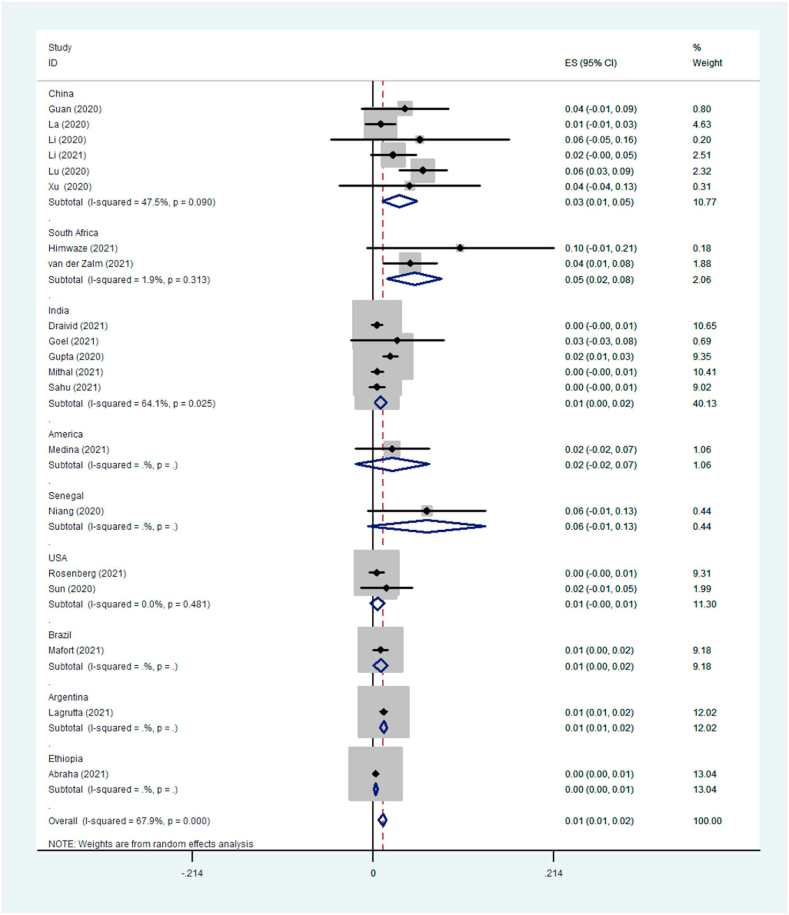
Forest plot of the meta-analysis on the prevalence of TB among patients with COVID-19 based on countries.

Eighteen prevalence studies were evaluated in the present review, six reported from China (33%), four from India (22%), two from the USA (11%), and Argentina, Brazil, Guatemala, Senegal, South Africa, and Zambia each reported one study (5%). These studies had 5843 participants with COVID-19, of which 101 patients had TB coinfection. The pooled prevalence of TB coinfection among patients with COVID-19 was 1.1% (95% CI: 67.9). The meta-analysis of prevalence studies revealed that the frequency of TB coinfection among patients with COVID-19 was 1.5% (95% CI 0.7–2.3) in Asia (10 studies, 50 patients), 3.6% (95% CI 0.2–7.5) in Africa (3 studies, 13 patients) and 1.1% (95% CI 0.7–1.4) from the America continent (5 studies, 38 patients). When this study was conducted, there were no reports of coinfection with TB in patients with COVID-19 from Europe or Oceania (Table 3). The related analysis included a forest plot, funnel plot, and Galbraith of the meta-analysis on the prevalence of TB among patients with COVID-19, provided in Fig. 2, Fig. 3, Fig. 4, Fig. 5, Fig. 6.

**Table 3:** Frequency of TB infection among patients with COVID-19 based on different subgroups.

patients with COVID-19 and TB		Prevalence% (95% CI)	Number of studies	Number of patients	I-squared
**Overall**		1.1 (0.7–1.6)	**18**	**101**	**67.9**
**Continent**	***America***	1.1 (0.7–1.4)	5	38	0.0%
	***Asia***	1.5 (0.7–2.3)	10	50	63.3%
	***Africa***	3.6 (−0.2-7.5)	3	13	75.5%
**Country**	***China***	3.1 (1.1–5.2)	6	24	47.5%
	***India***	0.9 (0.2–1.6)	4	26	64.1%
	***USA***	0.6 (−0.2–1.4)	2	2	0.0%

### 3.2 Case reports/case series studies

Eighteen case reports (96 patients) and 57 case series (101 patients) highlighted tuberculosis coinfection in 43 and 53 COVID-19 patients, respectively. Of these 96 patients (i.e., 22 female, 67 male, and seven were not reported), the population of men was almost three times that of women. Except for patients whose mean age was not reported (8 patients) and non-adult ones (4 patients), the mean age of adult patients was 45.14 years. Table 4 provides details about these studies; as can be seen, diabetes (15.62%) and hypertension (8.3%) were among the most prevalent comorbidities of coinfected patients. Chronic kidney disease, with a prevalence of 6.25%, was the third most common comorbidity in these patients. According to details regarding concurrent infections, HIV (11.45%) was the most common infection among coinfected patients. Clinical symptoms were also examined in COVID-19 and TB coinfected patients. Fever, cough, and weight loss were the most common clinical manifestations reported in this group of patients, with a frequency of 66.66, 56.25, and 22.91%, respectively. Based on the results of included studies, the real-time reverse transcription-polymerase chain reaction (RT-PCR) (56.25%), chest x-ray (CXR) (35.41%), and computed tomography (CT) scan (32.29%) were the most common diagnostic method for COVID-19. Acid-fast bacillus (AFB) testing (30.20%) was the most common diagnostic method for TB (Table 5). According to the laboratory findings reported in the evaluated articles, elevated *C*-reactive protein (CRP) (45.83%), lymphopenia (25%), and elevated lactate dehydrogenase (LDH) (25%) were the most commonly reported findings, respectively. Monocytosis was the least reported abnormality (Table 4). The type of tuberculosis was also evaluated in case reports/case series studies, of which 17 studies reported the active type and six reported the latent type. It was also found that the most common presentation of TB in coinfected patients was pulmonary involvement in 14 studies. The outcomes of COVID-19 and TB coinfection were reported in 52 studies. According to them, 20 of 96 patients died (20.83%), and 63 out of 96 patients recovered (65.62%) (Table 4). There were three treatment groups for patients with COVID-19 co-infected with TB, including antiviral drugs, antibacterial drugs, and a combination of drugs (Table 5). Lopinavir/Ritonavir was the most widely used antiviral drug reported in seven studies. Among the antibacterial drugs, isoniazid, rifampin, ethambutol, and pyrazinamide were more common antibiotics used in 36, 31, 30, and 29 studies, respectively. After these drugs, we can refer to azithromycin (28.12%). Among combination therapies, Hydroxychloroquine consumption was the most common way (27.08%). In the next rank, oxygen supplementation and anticoagulant treatment were among the most widely used methods, each with nine studies. It can be seen that many diagnostic methods were used for COVID-19 infection, but the most common methods were RT-PCR, CT scan, and CXR with 39, 22, and 17 studies, respectively. In our findings, serological tests were not common (3.12%). Regarding TB diagnostic methods, we found that acid-fast bacillus (AFB) testing, PCR, culture, and CT scan were the most common methods, with 30.20, 21.87, 17.70, and 14.58%, respectively (Table 5).

**Table 4:** Summary of the case reports/case series findings.

Overall				
**Types of study**	**Number of studies**	**Total patients with COVID-19**	**Total patients with COVID-19 and TB**	**n/N**^a^**(%)**
**Case report**	18	114	96	96/114 (84.21)
**Case series**	57	5843	101	101/5843 (1.72)
Comorbidities	**Variables**	**Number of studies**	**Number of patients with co-infection**	**n/N**^a^**(%)**
	Hypertension	7	8	8/96 (8.3)
	Diabetes	11	15	15/96 (15.62)
	Chronic kidney disease	6	6	6/96 (6.25)
	Anemia	4	5	5/96 (5.2)
	Chronic obstructive pulmonary disease (COPD)	2	2	2/96 (2.08)
	Bronchiectasis	2	3	3/96 (3.12)
	Esophageal cancer	2	2	2/96 (2.08)
	Bladder neoplasm	1	1	1/96 (1.04)
	Nonsmall-cell lung carcinoma	1	1	1/96 (1.04)
	Coronary artery perforation	1	1	1/96 (1.04)
	Advanced cirrhosis	1	1	1/96 (1.04)
	Chronic liver disease	1	1	1/96 (1.04)
	Exfoliative dermatitis	1	1	1/96 (1.04)
	Obesity	1	1	1/96 (1.04)
	Parkinson	1	1	1/96 (1.04)
Concurrent infection	HIV	10	11	11/96 (11.45)
	*Klebsiella pneumoniae*	1	1	1/96 (1.04)
	Pseudomonas sp.	1	1	1/96 (1.04)
	Stenotrophomonas sp.	1	1	1/96 (1.04)
	Trichosporon sp.	1	1	1/96 (1.04)
	CMV	1	1	1/96 (1.04)
	Aspergillus	1	1	1/96 (1.04)
Clinical manifestation	Fever	41	64	64/96 (66.66)
	Headache	10	10	10/96 (10.41)
	Diarrhea	3	5	5/96 (5.2)
	Emphysema	3	4	4/96 (4.16)
	Myalgia	9	11	11/96 (11.45)
	Fatigue	8	12	12.96 (12.5)
	Cough with sputum production	9	11	11/96 (11.45)
	Cough	36	54	54/96 (56.25)
	Lose appetite	4	9	9/96 (9.37)
	Decreased appetite	4	4	4/96 (4.16)
	Respiratory failure/distress	10	14	14/96 (14.58)
	Shortness of breath	11	12	12/96 (12.5)
	Reduction in breath sounds	3	5	5/96 (5.2)
	Dyspnea	14	16	16/96 (16.66)
	Hypoxia	15	16	16/96 (16.66)
	Chest pain	8	8	
	Abdominal pain	5	5	5/96 (5.2)
	Tachypnoea	8	8	8/96 (8.33)
	Tachycardia	6	6	6/96 (6.25)
	Night sweats	5	5	5/96 (5.2)
	Neurologic presentation	2	7	7/96 (7.29)
	Seizures	1	6	6/96 (6.25)
	Rash	2	3	3/96 (3.12)
	Wheeze	1	3	3/96 (3.12)
	Weakness	3	3	3/96 (3.12)
	Asthenia	2	3	3/96 (3.12)
	Chest tightness	2	3	3/96 (3.12)
	Pallor	2	3	3/96 (3.12)
	Weight loss	14	22	22/96 (22.91)
Laboratory finding	Neutrophilia	11	12	12.96 (12.5)
	Lymphopenia	18	24	24/96 [25]
	Monocytosis	4	4	4/96 (4.16)
	Leucopenia	5	7	7/96 (7.29)
	Leukocytosis	12	18	18/96 (18.75)
	Low hemoglobin	11	14	14/96 (14.58)
	Thrombocytopenia	3	5	5/96 (5.2)
	High platelets	4	5	5/96 (5.2)
	Low albumin	6	7	7/96 (7.29)
	High fibrinogen	5	7	7/96 (7.29)
	High ALT	6	10	10/96 (10.41)
	High AST	9	13	13/96 (13.54)
	High CRP	31	44	44/96 (45.83)
	High Procalcitonin	6	6	6/96 (6.25)
	High LDH	16	24	24/96 [25]
	High ferritin	13	14	14/96 (14.58)
	High ESR	10	16	16/96 (16.66)
	High D-dimer	16	21	21/96 (21.87)
	High interleukin-6	5	7	7/96 (7.29)
	High creatinine	6	7	7/96 (7.29)
Imaging	Bilateral pneumonia	2	6	6/96 (6.25)
	Unilateral pneumonia	2	2	2/96 (2.08)
	Interstitial involvement	4	4	4/96 (4.16)
	Pulmonary infiltrates	9	15	15/96 (15.62)
	Tree-in-bud opacities	3	7	7/96 (7.29)
	Centrilobular nodules	1	2	2/96 (2.08)
	Hilar lymph nodes	3	4	4/96 (4.16)
	Reticular pattern	2	2	2/96 (2.08)
	Patchy opacities/consoliation	5	9	9/96 (9.37)
	Patchy ground-glass opacities	4	6	6/96 (6.25)
	Consolidation	18	23	23/96 (23.95)
	Crazy pavement pattern	2	2	2/96 (2.08)
	Ground-glass opacity	13	15	15/96 (15.62)
	Mediastinal lymphadenopathy	3	4	4/96 (4.16)
	Atelectasis	4	4	4/96 (4.16)
	Cavitation	8	16	16/96 (16.66)
	Pleural effusion	9	12	12.96 (12.5)
Type of TB	Pulmonary TB	14	23	23/96 (23.95)
	Tuberculous meningitis	1	1	1/96 (1.04)
	Abdominopelvic tuberculosis	1	1	1/96 (1.04)
	Disseminated tuberculosis	2	2	2/96 (2.08)
	Parenchymal and endobronchial tuberculosis	1	1	1/96 (1.04)
	Lymphadenitis tuberculosis	1	1	1/96 (1.04)
	Active	17	29	29/96 (30.20)
	Latent	6	6	6/96 (6.25)
Outcome	Live	52	63	63/96 (65.62)
	Dead	52	20	20/96 (20.83)

a n, number of patients with any variables; N, the total number of patients with COVID-19 and TB, nr; not report.

**Table 5:** Agents used in the treatment of patients with COVID-19 and TB.

	Agent	Number of studies	Number of patients with co-infection	n/N* (%)
Antiviral drug	Remdesivir	2	2	2/96 (2.08)
	Lopinavir/ritonavir	7	10	10/96 (10.41)
	Sovodak (sofosbuvir/daclatasvir)	1	3	3/96 (3.12)
	Umifenovir	2	4	4/96 (4.16)
	Oseltamivir	1	1	1/96 (1.04)
	Favipiravir	1	1	1/96 (1.04)
	Valacyclovir	1	1	1/96 (1.04)
	Ganciclovir	1	1	1/96 (1.04)
	Ribavirin	1	1	1/96 (1.04)
Antibacterial drug	Levofloxacin	5	5	5/96 (5.2)
	Isoniazid	36	47	47/96 (48.95)
	Rifampicin	31	41	41/96 (42.70)
	Ethambutol	30	41	41/96 (42.70)
	Pyrazinamide	29	40	40/96 (41.66)
	Azithromycin	18	27	27/96 (28.12)
	Ceftriaxone	8	15	15/96 (15.62)
	Piperacillin-tazobactam	6	6	6/96 (6.25)
	Ampicillin	2	2	2/96 (2.08)
	Amoxicillin/clavulanic acid	2	2	2/96 (2.08)
	Ceftazidime	2	2	2/96 (2.08)
	Doxycycline	2	2	2/96 (2.08)
Combination therapy	Oxygen supplementation	9	10	10/96 (10.41)
	Respiratory support via a high flow nasal cannul	4	4	4/96 (4.16)
	Methyl prednisolone	5	6	6/96 (6.25)
	Corticosteroids	3	5	5/96 (5.2)
	Anticoagulants	9	10	10/96 (10.41)
	Zinc sulphate	2	3	3/96 (3.12)
	Pyridoxine (vitamin B₆)	3	4	4/96 (4.16)
	Vitamin C	3	3	3/96 (3.12)
	Hydroxychloroquine	18	26	26/96 (27.08)
	Dexamethasone	5	5	5/96 (5.2)
	Tocilizumab	4	4	4/96 (4.16)
	Chloroquine	3	3	3/96 (3.12)
	Metformin	2	2	2/96 (2.08)
	Convalescent plasma	3	3	3/96 (3.12)
	Interferon	2	2	2/96 (2.08)
COVID-19 diagnostic method	**Variables**	**Number of studies**	**Number of patients with co-infection**	n/N* (%)
	RT-PCR	39	54	54/96 (56.25)
	PCR	12	18	18/96 (18.75)
	Real-time fluorescence PCR	1	3	3/96 (3.12)
	Serological test	3	3	3/96 (3.12)
	CXR	17	34	34/96 (35.41)
	CT	22	31	31/96 (32.29)
TB diagnostic method	Acid-Fast Bacillus (AFB) Testing	14	29	29/96 (30.20)
	Culture	10	17	17/96 (17.70)
	RIF Assay	5	7	7/96 (7.29)
	Genexpert	3	10	10/96 (10.41)
	Xpert MTB/Rif	5	5	5/96 (5.2)
	CT scan	12	14	14/96 (14.58)
	HRCT	1	3	3/96 (3.12)
	Chest X ray	6	7	7/96 (7.29)
	Molecular testing	1	4	4/96 (4.16)
	PCR	10	21	21/96 (21.87)
	Interferon-gamma release assay (IGRA)	1	4	4/96 (4.16)

## 4 Discussion

COVID-19 is a detrimental respiratory infection that has become a global problem due to its high transmission and lethality [18]. As a result, many studies have been conducted to learn more about the disease's mechanisms of pathogenesis, symptoms, and complications. COVID-19 is currently an essential topic of research. However, coinfections with COVID-19 are also a significant concern because they can worsen the course of the disease [19]. Some of these concurrent infections are caused by bacteria. One essential bacteria is *M. tuberculosis*, which causes tuberculosis [20,21]. Tuberculosis is a contagious and deadly respiratory infection that killed 1.3 million people in 2020 (among HIV-negative people) [22]. China ranks third among the eight countries that account for two-thirds of TB cases worldwide, contributing 8.4% to the globally reported cases [23]. Over a quarter (27%) of all TB cases in the world are reported from India, which continues to have the highest burden of TB [24]. The 50 U S. states and the District of Columbia (DC) provisionally reported 7860 TB cases to the National Tuberculosis Surveillance System (NTSS) in 2021. In 2021, reported TB cases increased by 9.4% (2.37) compared to 2020 (2.16) [25]. Each year, approximately 9000 cases of TB are reported in Argentina. The disease's distribution is not uniform throughout the country. The number of incident TB cases in Zambia in 2019 was estimated at 59,000 (333 per 100,000). The TB burden in Zambia remains among the highest in the world, although TB trends have been declining steadily over the years [26]. South Africa ranks among the top 20 countries with high tuberculosis burden. As of 2018, there were 737 cases of TB per 100,000 in South Africa [27,28]. Brazil ranks 16th on the list of 22 countries with the highest TB burden globally. The state of Rio de Janeiro has the highest TB incidence rate (61.2/100,000) and the highest mortality rate (5.0/100,000) [29]. Therefore, tuberculosis infection in COVID-19 patients can be expected to aggravate the complications and increase disease mortality. In 2020, the Global Tuberculosis Network (GTN) published the results of a pilot study on 49 patients co-infected with both TB and COVID-19 [30]. The authors concluded that, although the symptoms and signs are similar, TB is commonly diagnosed with or after COVID-19, and dual infection may increase the mortality rate. According to a second GTN study [31] involving 69 TB/COVID-19 patients, the overall case-fatality rate was 12.6%, higher than the 1–2% rate reported for drug-susceptible TB [10] and COVID-19 [32]. Further studies from South Africa and the Philippines revealed that COVID-19 patients with TB have a 2.7 [33] and 2.17 [34] higher mortality risk compared to COVID-19 patients without TB. Common symptoms of COVID-19 include fever, cough, and shortness of breath, similar to symptoms of patients with tuberculosis. So, it is logical that overlapping similar symptoms in COVID-19 and tuberculosis coinfection can interfere with the diagnosis and treatment. Our study showed that fever, cough, and weight loss are the most common symptoms among COVID-19 and tuberculosis coinfected patients. Another study conducted in China reported similar results [35]. COVID-19 and tuberculosis are transmitted through droplets; their target organs are the lungs. Both of them stimulate T lymphocytes, especially helper T lymphocytes, by different mechanisms, which ultimately, in severe forms of these two diseases, lead to increased production and secretion of interferons [36]. So, it is likely that they can exacerbate each other's complications. In Wang's study, it was found that patients with COVID-19 and TB coinfection were at high risk of severity than other COVID-19 patients [37]. Due to these facts, tuberculosis was the leading cause of death among infectious diseases before the onset of COVID-19, and about 10 million people, regardless of gender or age, became infected in 2020 [38]. Scattered studies have been conducted worldwide on the simultaneous presence of these two infections. Since we do not have accurate statistics about this coinfection, we evaluated the relevant prevalence and case report/series studies through systematic review and meta-analysis. Our meta-analysis showed that 1.1% of COVID-19 patients simultaneously had tuberculosis infections. Similarly, Ashutosh et al. estimated that the active pulmonary tuberculosis pool proportion was 1.07% among patients with COVID-19. Based on their study, the mortality risk was higher in patients with COVID-19 and active pulmonary tuberculosis than in others [39]. Overall, the rate of coinfection with COVID-19 and tuberculosis has been reported between 0.6% and 3.6% in the reviewed study. To justify this dispersion and difference, we can point to various reasons, including the study population, other comorbidities of patients like diabetes or hypertension, the methods of diagnosis, and the time and place of investigation regarding the prevalence of tuberculosis. Tuberculosis coinfection among patients with COVID-19 was more reported in Africa and China. Since Africa has poor health care and tuberculosis was prevalent in Africa before the advent of COVID-19 [40], it is reasonable to be likely that patients with COVID-19 and tuberculosis be more prevalent in this region. In the case of China, the high population and frequent passenger traffic may contribute to these statistics. Based on evaluated case reports/series studies, we examined other tuberculosis disorders and pulmonary involvement. The investigated studies showed that six people had extrapulmonary tuberculosis, five of whom survived. Nevertheless, due to the minimal number of such cases and lack of access to accurate information worldwide, we could not figure out the exact association of COVID-19 disease with non-pulmonary tuberculosis. One of the highlights of our study was that among concurrent infections, HIV accounted for the highest percentage. Since tuberculosis is an opportunistic infection and people with HIV have weakened immune systems than others, it is reasonable to see more tuberculosis and HIV coinfected patients [41]. According to our study, among the medications used in patients with simultaneous COVID-19 and tuberculosis, the highest consumption belonged to medicines for treating tuberculosis. There is no definitive known cure for all COVID-19 patients. Still, unlike COVID-19, tuberculosis has a specific treatment, so the higher usage of anti-tuberculosis drugs in patients with COVID-19 and TB coinfection can be justified. Our results also showed that using azithromycin and hydroxychloroquine is significant among COVID-19 and TB coinfected patients. Nevertheless, it should be noted that according to recent studies, hydroxychloroquine does not have much effect on the progression of COVID-19, and there is controversy about the effectiveness of azithromycin [42,43]. Many studies were reviewed on the early onset of tuberculosis, and at that time, various drugs were used to treat patients. Therefore, the high percentage of azithromycin and hydroxychloroquine consumption can be related to this issue. Our study had several limitations. First, we did not have enough information from many countries, so we could not fully demonstrate the prevalence of TB infection in COVID-19 patients worldwide. Second, many COVID-19 patients with TB may not have been hospitalized. Third, we only included studies published in English in our research. Fourth, some reviewed studies did not mention the type of TB. Finally, since the generality of the current study is based on the existing articles and the information provided, it was impossible to access detailed information in this case.

## 5 Conclusion

The simultaneous prevalence of tuberculosis among patients with COVID-19 was 1.1% in general. According to the investigations, it was found that the simultaneous infection of tuberculosis and COVID-19 has been observed in Africa, Asia, and America. Furthermore, TB infection may also increase mortality in COVID-19 patients. Based on different evaluated studies, various drug treatments were performed in COVID-19 patients with tuberculosis, and antibacterial drugs were the most used. The smart selection of the medication and faster diagnostic methods can play an essential role in the recovery of these patients. Since symptoms of TB and COVID-19 overlap, screening TB patients to rule out COVID-19 infection is highly recommended to prevent further damage in this group of patients. It would be helpful to perform further research on the relationship between COVID-19 and extrapulmonary TB coinfections.

## Author contribution statement

All authors listed have significantly contributed to the development and the writing of this article. </p>

## Funding statement

This research did not receive any specific grant from funding agencies in the public, commercial, or not-for-profit sectors.

## Data availability statement

Data included in article/supp. Material/referenced in article.

## Declaration of interest's statement

The authors declare that there are no potential conflicts of interest in the present study.

## Additional information

No additional information is available for this paper.
